# Endocannabinoids: A Promising Impact for Traumatic Brain Injury

**DOI:** 10.3389/fphar.2017.00069

**Published:** 2017-02-17

**Authors:** Lesley D. Schurman, Aron H. Lichtman

**Affiliations:** Department of Pharmacology and Toxicology, Virginia Commonwealth University, RichmondVA, USA

**Keywords:** traumatic brain injury, cannabinoid, endocannabinoid, neuroprotection, phytocannabinoid

## Abstract

The endogenous cannabinoid (endocannabinoid) system regulates a diverse array of physiological processes and unsurprisingly possesses considerable potential targets for the potential treatment of numerous disease states, including two receptors (i.e., CB_1_ and CB_2_ receptors) and enzymes regulating their endogenous ligands *N*-arachidonoylethanolamine (anandamide) and 2-arachidonyl glycerol (2-AG). Increases in brain levels of endocannabinoids to pathogenic events suggest this system plays a role in compensatory repair mechanisms. Traumatic brain injury (TBI) pathology remains mostly refractory to currently available drugs, perhaps due to its heterogeneous nature in etiology, clinical presentation, and severity. Here, we review pre-clinical studies assessing the therapeutic potential of cannabinoids and manipulations of the endocannabinoid system to ameliorate TBI pathology. Specifically, manipulations of endocannabinoid degradative enzymes (e.g., fatty acid amide hydrolase, monoacylglycerol lipase, and α/β-hydrolase domain-6), CB_1_ and CB_2_ receptors, and their endogenous ligands have shown promise in modulating cellular and molecular hallmarks of TBI pathology such as; cell death, excitotoxicity, neuroinflammation, cerebrovascular breakdown, and cell structure and remodeling. TBI-induced behavioral deficits, such as learning and memory, neurological motor impairments, post-traumatic convulsions or seizures, and anxiety also respond to manipulations of the endocannabinoid system. As such, the endocannabinoid system possesses potential drugable receptor and enzyme targets for the treatment of diverse TBI pathology. Yet, full characterization of TBI-induced changes in endocannabinoid ligands, enzymes, and receptor populations will be important to understand that role this system plays in TBI pathology. Promising classes of compounds, such as the plant-derived phytocannabinoids, synthetic cannabinoids, and endocannabinoids, as well as their non-cannabinoid receptor targets, such as TRPV1 receptors, represent important areas of basic research and potential therapeutic interest to treat TBI.

## Introduction

Traumatic brain injury accounts for approximately 10 million deaths and/or hospitalizations annually in the world, and approximately 1.5 million annual emergency room visits and hospitalizations in the US (Langlois et al., 2006). Young men are consistently over-represented as being at greatest risk for TBI (Langlois et al., 2006). While half of all traumatic deaths in the USA are due to brain injury (Mayer and Badjatia, 2010), the majority of head injuries are considered mild and often never receive medical treatment (Corrigan et al., 2010). Survivors of TBI are at risk for lowered life expectancy, dying at a 3⋅2 times more rapid rate than the general population (Baguley et al., 2012). Survivors also face long term physical, cognitive, and psychological disorders that greatly diminish quality of life. Even so-called mild TBI without notable cell death may lead to enduring cognitive deficits (Niogi et al., 2008; Rubovitch et al., 2011). A 2007 study estimated that TBI results in $330,827 of average lifetime costs associated with disability and lost productivity, and greatly outweighs the $65,504 estimated costs for initial medical care and rehabilitation (Faul et al., 2007), demonstrating both the long term financial and human toll of TBI.

The development of management protocols in major trauma centers (Brain Trauma Foundation et al., 2007) has improved mortality and functional outcomes (Stein et al., 2010). Monitoring of intracranial pressure is now standard practice (Bratton et al., 2007), and advanced MRI technologies help define the extent of brain injury in some cases (Shah et al., 2012). Current treatment of major TBI is primarily managed through surgical intervention by decompressive craniotomy (Bullock et al., 2006) which involves the removal of skull segments to reduce intracranial pressure. Delayed decompressive craniotomy is also increasingly used for intractable intracranial hypertension (Sahuquillo and Arikan, 2006). The craniotomy procedure is associated with considerable complications, such as hematoma, subdural hygroma, and hydrocephalus (Stiver, 2009). At present, the pathology associated with TBI remains refractive to currently available pharmacotherapies (Meyer et al., 2010) and as such represents an area of great research interest and in need of new potential targets. Effective TBI drug therapies have yet to be proven, despite promising preclinical data (Lu et al., 2007; Mbye et al., 2009; Sen and Gulati, 2010) plagued by translational problems once reaching clinical trials (Temkin et al., 2007; Tapia-Perez et al., 2008; Mazzeo et al., 2009).

The many biochemical events that occur in the hours and months following TBI have yielded preclinical studies directed toward a single injury mechanism. However, an underlying premise of the present review is an important need to address the multiple targets associated with secondary injury cascades following TBI. A growing body of published scientific research indicates that the endogenous cannabinoid (endocannabinoid; eCB) system possesses several targets uniquely positioned to modulate several key secondary events associated with TBI. Here, we review the preclinical work examining the roles that the different components of the eCB system play in ameliorating pathologies associated with TBI.

## The Endocannabinoid (eCB) System

Originally, “Cannabinoid” was the collective name assigned to the set of naturally occurring aromatic hydrocarbon compounds in the *Cannabis sativa* plant (Mechoulam and Goani, 1967). Cannabinoid now more generally refers to a much more broad set of chemicals of diverse structure whose pharmacological actions or structure closely mimic that of plant-derived cannabinoids. Three predominant categories are currently in use; plant-derived phytocannabinoids (reviewed in Gertsch et al., 2010), synthetically produced cannabinoids used as research (Wiley et al., 2014) or recreational drugs (Mills et al., 2015), and the endogenous cannabinoids, *N*-arachidonoylethanolamine (anandamide) (Devane et al., 1992) and 2-AG (Mechoulam et al., 1995; Sugiura et al., 1995).

These three broad categories of cannabinoids generally act through cannabinoid receptors, two types of which have so far been identified, CB_1_ (Devane et al., 1988) and CB_2_ (Munro et al., 1993). Both CB_1_ and CB_2_ receptors are coupled to signaling cascades predominantly through G_i/o_-coupled proteins. CB_1_ receptors mediate most of the psychomimetic effects of cannabis, its chief psychoactive constituent THC, and many other CNS active cannabinoids. These receptors are predominantly expressed on pre-synaptic axon terminals (Alger and Kim, 2011), are activated by endogenous cannabinoids that function as retrograde messengers, which are released from post-synaptic cells, and their activation ultimately dampens pre-synaptic neurotransmitter release (Mackie, 2006). Acting as a neuromodulatory network, the outcome of cannabinoid receptor signaling depends on cell type and location. CB_1_ receptors are highly expressed on neurons in the central nervous system (CNS) in areas such as cerebral cortex, hippocampus, caudate-putamen (Herkenham et al., 1991). In contrast, CB_2_ receptors are predominantly expressed on immune cells, microglia in the CNS, and macrophages, monocytes, CD4+ and CD8+ T cells, and B cells in the periphery (Cabral et al., 2008). Additionally, CB_2_ receptors are expressed on neurons, but to a much less extent than CB_1_ receptors (Atwood and MacKie, 2010). The abundant, yet heterogeneous, distribution of CB_1_ and CB_2_ receptors throughout the brain and periphery likely accounts for their ability to impact a wide variety of physiological and psychological processes (e.g., memory, anxiety, and pain perception, reviewed in Di Marzo, 2008) many of which are impacted following TBI.

Another unique property of the eCB system is the functional selectivity produced by its endogenous ligands. Traditional neurotransmitter systems elicit differential activation of signaling pathways through activation of receptor subtypes by one neurotransmitter (Siegel, 1999). However, it is the endogenous ligands of eCB receptors which produce such signaling specificity. Although several endogenous cannabinoids have been described (Porter et al., 2002; Chu et al., 2003; Heimann et al., 2007) the two most studied are anandamide (Devane et al., 1992) and 2-AG (Mechoulam et al., 1995; Sugiura et al., 1995). 2-AG levels are three orders of magnitude higher than those of anandamide in brain (Béquet et al., 2007). Additionally, their receptor affinity (Pertwee and Ross, 2002; Reggio, 2002) and efficacy differ, with 2-AG acting as a high efficacy agonist at CB_1_ and CB_2_ receptors, while anandamide behaves as a partial agonist (Hillard, 2000a). In addition, anandamide binds and activates TRPV1 receptors (Melck et al., 1999; Zygmunt et al., 1999; Smart et al., 2000), whereas 2-AG also binds GABA_A_ receptors (Sigel et al., 2011). As such, cannabinoid ligands differentially modulate similar physiological and pathological processes.

**FIGURE 1:**
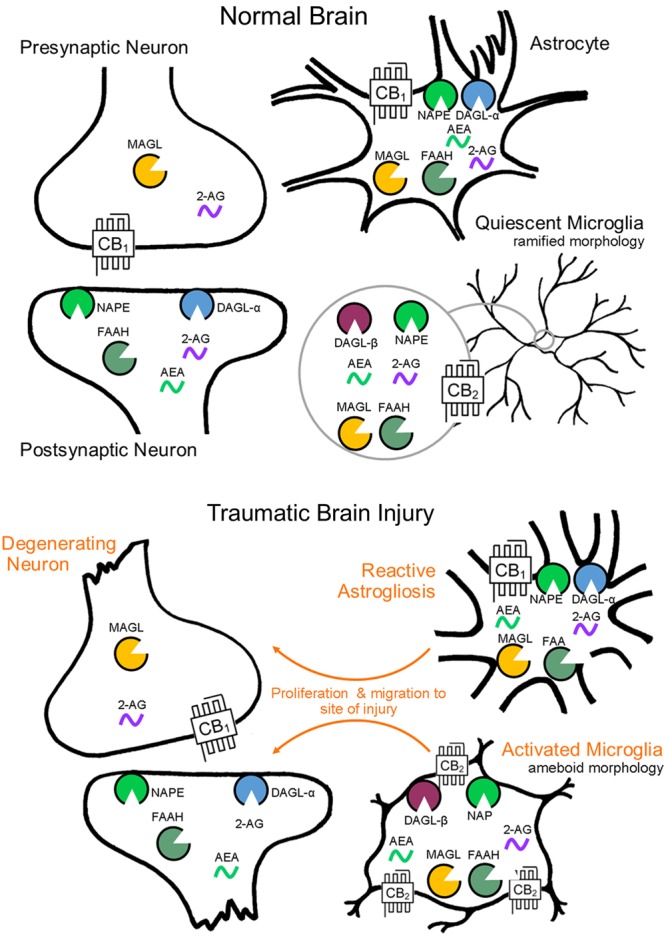
**Endocannabinoid system cell localization by CNS cell type.** Endocannabinoid functional specialization among CNS cell types is determined by the cellular compartmentalization of biosynthetic and catabolic enzymes (biosynthesis by NAPE and DAGL-α, -β, catabolism by FAAH and MAGL). Cellular level changes in eCB biosynthetic and catabolic enzymes as a result of brain injury have yet to be investigated, though morphological and molecular reactivity by cell type is well documented.

Distinct sets of enzymes, which regulate the biosynthesis and degradation of the eCBs and possess distinct anatomical distributions (see **Figure 1**), exert control over CB_1_ and CB_2_ receptor signaling. Inactivation of anandamide occurs predominantly through FAAH (Cravatt et al., 1996, 2001), localized to intracellular membranes of postsynaptic somata and dendrites (Gulyas et al., 2004), in areas such as the neocortex, cerebellar cortex, and hippocampus (Egertová et al., 1998). Inactivation of 2-AG proceeds primarily via MAGL (Dinh et al., 2002; Blankman et al., 2007), expressed on presynaptic axon terminals (Gulyas et al., 2004), and demonstrates highest expression in areas such as the thalamus, hippocampus, cortex, and cerebellum (Dinh et al., 2002). The availability of pharmacological inhibitors for eCB catabolic enzymes has allowed the selective amplification of anandamide and 2-AG levels following brain injury as a key strategy to enhance eCB signaling and to investigate their potential neuroprotective effects.

**FIGURE 2:**
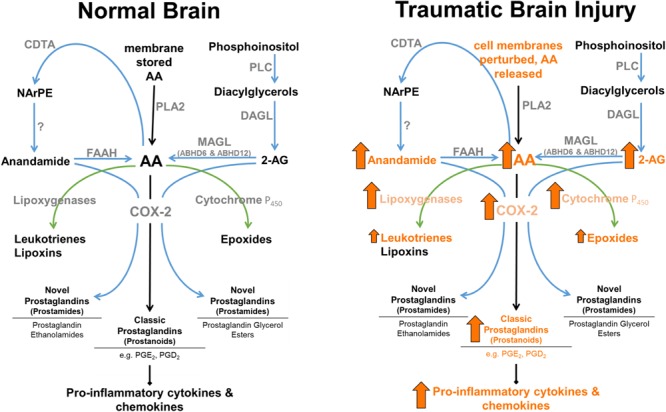
**Enzymatic regulation of anandamide and 2-AG in normal brain, and following TBI.** 2-AG levels are approximately 1,000-fold higher than anandamide levels in brain. MAGL plays a rate-limited role in the production of AA in brain, lung, and liver (Nomura et al., 2008). Arrows represent known TBI-induced changes in eCBs, catabolic and downstream enzymes, and their metabolic products (arrow size has no relation to magnitude of change).

Finally, 2-AG functions not only as a major cannabinoid receptor signaling molecule, but also serves as a major precursor for AA, and therefore plays a role in inflammatory pathways (see **Figure 2**). Although AA is a degradative product of both 2-AG (Bell et al., 1979) and anandamide (Deutsch et al., 1997), MAGL represents a rate-limiting biosynthetic enzyme of highly bioactive lipid in brain, liver, and lung (Nomura et al., 2011). Historically, cPLA2 was considered to be the primary rate-limiting enzyme in AA production (reviewed in Buczynski et al., 2009). However, MAGL contributes ∼80% and cPLA2 ∼20% of LPS-stimulated eicosanoids in mouse brain. In contrast, cPLA2 is the dominant enzyme to control AA production in spleen (Nomura et al., 2011). Therefore, MAGL and cPLA2 appear to play differential roles in AA production, and concomitantly its eicosanoid metabolites in a tissue-specific manner (Nomura et al., 2011). As such, 2-AG functions not only as an endogenous CB1 and CB2 receptor ligand, but also an immunomodulator by virtue of its being a major precursor for AA, making it a versatile target for the treatment of TBI related pro-inflammatory pathologies. Understanding the biosynthesis mechanisms of eCBs may prove useful in modulating their entry into pro-inflammatory pathways. While 2-AG is known to be synthesized by DAGL-α and DAGL-β (Bisogno et al., 2003), the mechanisms mediating anandamide production are incompletely understood (Blankman and Cravatt, 2013).

## Traumatic Brain Injury Pathology

Traumatic brain injuries are heterogeneous in their etiology, clinical presentation, severity, and pathology. The sequelae of molecular, biochemical, and physiological events that follow the application of an external mechanical force produce interacting acute and delayed pathologies, described as primary and secondary injuries. The initial insult produces an immediate mechanical disruption of brain tissue (Reilly, 2001). This primary injury consists of contusion, blood vessel disruption and brain oedema, localized necrotic cell death, as well as diffuse axonal injury producing degeneration of cerebral white matter (Adams et al., 1989; Gaetz, 2004).

Secondary injury mechanisms are initiated within minutes, in which necrotic and apoptotic cell death in contused areas and pericontusional penumbra continue over a period of days to months (Raghupathi, 2004). Neuronal disruption spills excitatory amino acids into the interstitial space, producing glutamate-mediated excitotoxicity (Bullock et al., 1998). Massive influx of Ca^2+^ into cells (Floyd et al., 2010) produce mitochondrial dysfunction and the release of ROS which lead to further apoptosis (Zhao et al., 2005). Injury-induced activation of CNS resident glial cells, microglia, as well as recruitment of circulating inflammatory cells, e.g., macrophages, then produce secretion of inflammatory mediators, cytokines and chemokines (reviewed in Woodcock and Morganti-Kossmann, 2013). Increased intracranial pressure leads to reductions in cerebral blood flow (Shiina et al., 1998), while injury-induced breakdown of the cerebrovascular endothelium contributes to dysfunction of the BBB (Chodobski et al., 2012). Extracranial pathologies are also evident following TBI with pulmonary complications being the most common (Pelosi et al., 2005). NPE often develops early after brain injury, producing hypoxemia and further aggravating secondary brain injury (Brambrink and Dick, 1997; Oddo et al., 2010). These varied and interacting disease processes highlight the necessity to address the multiple targets associated with secondary injury cascades following TBI.

While there are many types of CNS injury models [e.g., spinal cord injury, lesion studies, focal and global ischemic injury etc. (Arai and Lo, 2009; Titomanlio et al., 2015)], this review will focus primarily on the work investigating manipulations of the eCB system in preclinical models of TBI.

## Pre-Clinical Evaluation of Cannabinoids to Treat TBI

While basal anandamide and 2-AG levels differ within various structures in the CNS, levels increase on demand in response to a given stimuli [e.g., the induction of nausea (Sticht et al., 2016) or pain states (Costa et al., 2008)]. eCBs are lipid messengers not stored in synaptic vesicles (likely due to their hydrophobicity) but rather synthesized in an activity-dependent manner from membrane phospholipid precursors (Alger and Kim, 2011). Consequently, eCB signaling is enhanced by a stimulus-response synthesis and release mechanism.

Endocannabinoid levels increase in selected CNS tissue following neuronal damage, which may reflect a self-neuroprotective response. NMDA excitotoxicity produces elevations of anandamide in ipsilateral cortex of rats by 4-fold at 4 h and 14-fold at 24 h, but with no changes in 2-AG levels (Hansen et al., 2002). Concussive head trauma in rats produces a similar pattern of findings in which modest increases of anandamide levels occur in ipsilateral cortex, and again with no change in 2-AG levels (Hansen et al., 2002). This pattern was replicated by Tchantchou et al. (2014), who found a 1.5-fold increase of anandamide levels at 3 days post-TBI in ipsilateral mouse brain, and with no change in 2-AG. In contrast, Panikashvili et al. (2001) reported that TBI in mice led to increases of 2-AG in ipsilateral brain from 1 to 24 h with elevations as high as 10-fold. Thus, further research is needed to discern whether species differences, the model used to elicit neurotrauma, and/or other procedural considerations contribute to the differential elevation of these eCBs (Mechoulam and Lichtman, 2003).

A lack of studies systematically investigating the consequences of TBI on changes in eCB levels in specific brain regions perhaps point to the difficulty in measuring changes in the volatile eCBs, prone to rapid degradation (Deutsch and Chin, 1993; Dinh et al., 2002). While pharmacological and genetic manipulations of the eCB system continue to be evaluated following TBI; full characterization of how eCB biosynthetic and degradative enzymes, receptors, and endogenous ligands, their precursors and catabolic products, change as a consequence of TBI remains to be fully illuminated.

### Treatment of Cellular and Molecular Pathophysiology of TBI

In this section, we review preclinical studies of cannabinoids in the context of their potential to protect against cellular and molecular TBI pathology (see **Table 1**).

**Table 1:** Effect of cannabinoids on TBI-induced cellular and molecular pathophysiology.

Compound/mutant	Dose	Species	TBI model/severity	Effect	Receptor mediated	Reference
**CNS cell death**						
O-1966	5 mg/kg, i.p.	Mouse C57BL/6	CCI, moderate	↓ Neurodegeneration	CB_2_	Amenta et al., 2012
PF3845	5 mg/kg, i.p.	Mouse C57BL/6	CCI, severe	↓ Lesion volume ↓ Neurodegeneration → Bcl-2, Hsp70 and 72	Not evaluated	Tchantchou et al., 2014
JZL184	10 mg/kg, i.p.	Mouse C57BL/6	CHI, mild repetitive	↓ Neurodegeneration	Not evaluated	Zhang et al., 2014
WWL70	10 mg/kg, i.p.	Mouse C57BL/6	CCI, severe	↓ Lesion volume ↓ Neurodegeneration	CB_1_ CB_1_ and CB_2_	Tchantchou and Zhang, 2013
**Excitotoxicity**						
Rimonabant	2 mg/kg, i.p.	Rat Sprague–Dawley	Lateral FPI, severe	mGluR_5_ receptor recovery at 6 weeks (no impact on mGluR_1_)	CB_1_	Wang et al., 2016
2-AG	5 mg/kg, i.p.	Mouse Sabra	CHI, severe	→ Levels of weak antioxidants	Not evaluated	Panikashvili et al., 2006
JZL184	10 mg/kg, i.p. 16 mg/kg, i.p.	Mouse C57BL/6 Rat W istar	CHI, mild repetitive Lateral FPI, mild	Glutamate receptor recovery Injury-induced ↓ in LTP protection GluA1 expression protection Injury-induced → in EPSC protection	Not evaluated Not evaluated	Zhang et al., 2014 Mayeux et al., 2016
**Neuroinflammation**						
CB^1^ -/-	N/A	Mouse C57BL/6	CHI, severe	No effect on NF-κB transactivation	N/A	Panikashvili et al., 2005
CB^1^ -/- +2-AG	N/A	Mouse C57BL/6	CHI, severe	No effect on NF-κB transactivation	N/A	Panikashvili et al., 2005
O-1966	5 mg/kg, i.p.	Mouse C57BL/6	CCI, moderate	Microglial activation protection	CB_2_	Amenta et al., 2012
PF3845	5 mg/kg, i.p.	Mouse C57BL/6	CCI, severe	↓ COX-2 Expression ↓ iNos expression	Not evaluated	Tchantchou et al., 2014
URB597	0.3 mg/kg, i.p.	Rat Sprague–Dawley	Lateral FPI, mild	Microglial activation protection	Not evaluated	Katz et al., 2015
2-AG	5 mg/kg, i.p. 5 mg/kg, i.p.	Mouse Sabra mouse C57BL/6	CHI, severe CHI, severe	↓ TNFα mRNA ↓ IL-1β mRNA ↓ IL-6 mRNA ↓ NF-κB translocation and transactivation	Not evaluated CB_1_	Panikashvili et al., 2006 Panikashvili et al., 2005
JZL184	10 mg/kg, i.p. 16 mg/kg, i.p.	Mouse C57BL/6 rat Sprague–Dawley	CHI, mild repetitive Lateral FPI, mild	↓ TNFα mRNA Microglial activation protection Microglial activation protection	Not evaluated Not evaluated	Zhang et al., 2014 Katz et al., 2015
WWL70	10 mg/kg, i.p.	Mouse C57BL/6	CCI, severe	↓ COX-2 expression ↓ iNos expression M1 to M2 phenotype	Not evaluated	Tchantchou and Zhang, 2013
**Cerebrovascular breakdown**						
URB597	0.3 mg/kg, i.p.	Rat Sprague–Dawley	Lateral FPI, mild	BBB integrity protection	Not evaluated	Katz et al., 2015
2-AG	5 mg/kg, i.p.	Mouse Sabra	CHI, severe	BBB integrity protection	Not evaluated	Panikashvili et al., 2006
JZL184	16 mg/kg, i.p.	Rat Sprague–Dawley	Lateral FPI, mild	BBB integrity protection	Not evaluated	Katz et al., 2015
WWL70	10 mg/kg, i.p.	Mouse C57BL/6	CCI, severe	BBB integrity protection	Not evaluated	Tchantchou and Zhang, 2013
**CNS cellular structure/remodeling**						
Vehicle (saline-5% ETOH)	4 μL	Rat Wistar	CHI, moderate	Diurnal CB_1_ expression abolished → Contralateral CB_1_ and CB^2^ expression		Martinez-Vargas et al., 2013
CB^1^ -/-	N/A	Mouse C57BL/6	CHI, severe	No effect on oedema	N/A	Panikashvili et al., 2005
CB^1^ -/- +2-AG	N/A	Mouse C57BL/6	CHI, severe	No effect on oedema	N/A	Panikashvili et al., 2005
Rimonabant	2 mg/kg, i.p.	Rat Sprague–Dawley	Lateral FPI, severe	↓ CB_1_ Expression at 6 weeks post-TBI	CB_1_	Wang et al., 2016
PF3845	5 mg/kg, i.p.	Mouse C57BL/6	CCI, severe	↓ APP → Synaptophysin	Not evaluated	Tchantchou et al., 2014
2-AG	5 mg/kg, i.v. 5 mg/kg, i.p.	Mouse Sabra Mouse C57BL/6	CHI, severe CHI, severe	↓ CA3 neuron loss Oedema protection Oedema protection	CB_1_ CB_1_	Panikashvili et al., 2001 Panikashvili et al., 2005
JZL184	10 mg/kg, i.p. 16 mg/kg, i.p.	Mouse C57BL/6 Rat Sprague-Dawley	CHI, mild repetitive Lateral FPI, mild	↓ APP ↓ Amyloid-β peptide ↓ TDP-43 and p-tau ↓ astrocyte activation	Not evaluated Not evaluated	Zhang et al., 2014 Mayeux et al., 2016
WWL70	10 mg/kg, i.p.	Mouse C57BL/6	CCI, severe	→ CB_1_ and CB_2_ Expression	Not evaluated	Tchantchou and Zhang, 2013

*Drug targets; CB_2_ receptor agonist (O-1966), FAAH inhibitor (PF3845), MAGL inhibitors (JZL184 and URB597), ABHD6 inhibitor (WWL70), and CB_1_ receptor antagonist (Rimonabant). TBI Models; CCI (controlled cortical impact), CHI (closed head injury), and FPI (fluid percussion injury).*

#### CNS Cell Death

Traumatic brain injury-induced neuronal loss occurs almost immediately as necrotic cell death and continues for months following the initial insult via both necrotic and apoptotic cell death (Raghupathi, 2004). From a traumatic insult, the initial contused area forms a regional primary lesion or infarct surrounding which is the pericontusional penumbra, the area immediately adjacent to the primary lesion and at risk for further neurodegeneration. The evolution of the pericontusional penumbra occurs largely due to secondary injury mechanisms and has long been considered a candidate for interventions to protect against, or salvage from, further injury (Wang et al., 2014). The investigation of cannabinoids on traumatic CNS cell death have thus far demonstrated efficacy in two areas; attenuated neurodegeneration and reduced lesion volume.

Neurodegeneration, commonly measured by reductions in the neuronal marker fluoro-jade C, has been found to be readily attenuated in mice by CB_2_ receptor agonists (Amenta et al., 2012), as well as by inhibitors of FAAH (Tchantchou et al., 2014) and MAGL (Zhang et al., 2014). Additionally, FAAH inhibitors produce reductions in lesion volume, and increased production of the heat shock proteins Hsp70, known to be structurally protective, and Hsp72, a negative regulator of apoptosis (Tchantchou et al., 2014). Tchantchou et al. (2014) also showed that FAAH inhibition increased expression of the anti-apoptotic protein Bcl-2.

Several enzymes hydrolyze 2-AG including MAGL, which accounts for an estimated 85% of its total hydrolysis, as well as ABHD6 and ABHD12, which are responsible for much of the remaining 15% (Blankman et al., 2007). Tchantchou and Zhang (2013) found that inhibition of ABHD6 also reduced lesion volume and lowered neurodegeneration in a mouse CCI model. A CB_1_ receptor antagonist attenuated the protective effects on lesion volume, while CB_1_ and CB_2_ receptor antagonists prevented the protective effects on neurodegeneration (Tchantchou and Zhang, 2013).

Combined, this evidence suggests that inhibitors of eCB hydrolysis offer protection against TBI-induced cell death which involve CB_1_ and CB_2_ receptors, though the distinction between the eCBs remains to be clarified. Few studies have evaluated interactions between anandamide and 2-AG in laboratory models of TBI. One study using a model of cerebral focal ischemia found that exogenously administered anandamide and 2-AG in combination reduced infarct size in rats, but with no facilitatory effects beyond anandamide or 2-AG alone (Wang et al., 2009). Given the recent availability of dual FAAH/MAGL inhibitors (Long et al., 2009; Niphakis et al., 2012), simultaneous blockade of these enzymes following TBI may further reveal some insight into the relationship between anandamide and 2-AG on TBI-induced cell death.

#### Excitotoxicity

Previous efforts to attenuate the effects of excitotoxicity following brain injury focused on NMDA receptor antagonists, presumably with the understanding that the induction of depressed NMDA receptor function would counteract TBI-induced excitotoxicity. This class of drugs showed promise in laboratory animal models of TBI (Shohami et al., 1995), but failed to produce long-term beneficial outcomes in clinical trials, despite some acute benefits of improved intracranial pressure and cerebral perfusion pressure (Knoller et al., 2002; Maas et al., 2006). Research investigating manipulations of the eCB system on glutamatergic functioning following TBI have thus far focused primarily on 2-AG, and paradoxically, its effectiveness to protect the integrity of glutamate receptor function.

Several studies investigating the effects of cannabinoids in laboratory animal models of TBI have focused on expression changes of metabotropic (mGluRbbb, mGluR_5_), AMPA (GluA1, GluA2), and NMDA (GluN1, GluN2A, GluN2B) glutamatergic receptors. Specifically, post-injury administration of the MAGL inhibitor JZL184 reversed TBI-induced reductions of GluN2A, GluN2B, and GluA1 receptor expression, but with no impact on GluN1 or GluA2 receptors (Zhang et al., 2014). The CB_1_ receptor antagonist Rimonabant did not alter injury-induced lowered expression of mGluRbbb, but surprisingly reversed reduced mGluR_5_ receptor expression 6 weeks following TBI (Wang et al., 2016). Both findings were completed 30 days post injury (Zhang et al., 2014; Wang et al., 2016), suggesting long term changes in glutamatergic function following acute administration of cannabinoids post-injury. However, little overlap is found between receptor expression endpoints across papers. In an example of contradictory patterns of GluA1 expression after injury, GluA1 expression was reduced in a study that subjected mice to a daily mild CHI on three consecutive days (Zhang et al., 2014), and was increased in rats subjected to a single lateral fluid percussion brain injury (Mayeux et al., 2016). In these studies, MAGL inhibition ameliorated both the reduced (Zhang et al., 2014) and increased (Mayeux et al., 2016) GluA1 expression. As discussed above (see Pre-Clinical Evaluation of Cannabinoids to Treat TBI), systematic investigation of species (mice vs. rat), brain injury model, number of injuries, and other experimental variables are needed to understand the consequences of brain injury on glutamate receptor changes.

Endocannabinoids are known to depress glutamate release from pre-synaptic terminals, and in particular, 2-AG has been explored in its ability to influence the functioning of electrochemical neurotransmission. MAGL inhibition has been found to protect against injury-induced increases in frequency and amplitude of EPSC in pyramidal neurons at the site of injury (Mayeux et al., 2016), which may suggest changes in pre-synaptic transmitter release or post-synaptic strength (Zhang et al., 2005). MAGL inhibition has also protected against injury-induced LTP impairments at hippocampal CA3–CA1 synapses (Zhang et al., 2014), implicating the restoration of glutamate receptor function in protection against TBI-induced memory impairments.

Finally, the excitotoxicity resulting from TBI is part of the sequelae of events that lead to release of damaging ROS. Antioxidants are known to prevent oxidation of free radicals and thus protects against the cellular damage in response to sudden ROS elevation. eCBs have been linked to the neuroprotective production of antioxidants as the administration of exogenous 2-AG following injury has been found to increase levels of antioxidants (Panikashvili et al., 2006).

Combined, these data suggest that MAGL represents a promising target to reduce the damaging effects of injury-induced excitotoxicity through complimentary molecular pathways.

#### Neuroinflammation

Hydrolytic enzymes of anandamide and 2-AG produce a shared metabolic product in the formation of free AA, the major substrate of the biosynthetic enzymes of pro-inflammatory eicosanoids (Nomura et al., 2011). Therefore, eCB oxidation not only produces inactivation at cannabinoid receptors, but also leads to the production of bioactive lipids involved in inflammatory responses during the early stages of injury. Manipulations of the eCB system have proved effective in downregulating inflammation in many experimental models, such as inflammatory pain (Ahn et al., 2009), and multiple sclerosis (Mestre et al., 2005). The use of cannabinoids following TBI have thus far been linked to two predominant features of inflammation; decreased inflammatory cell activation, and decreases in pro-inflammatory cytokine production.

Pro-inflammatory activated microglia are known to exacerbate TBI-induced neuroinflammation (Kigerl et al., 2009). Thus, decreasing TBI-inductions of inflammatory cell activation is an attractive treatment strategy. MAGL inhibition protects against TBI-induced microglial activation (Zhang et al., 2014; Katz et al., 2015), while ABHD6 inhibition promotes microglia/macrophage shift from a pro-inflammatory M1 to an anti-inflammatory M2 phenotype (Tchantchou and Zhang, 2013). A parsimonious explanation for these findings is that prevention of 2-AG hydrolysis leads to reduced levels of AA and concomitant reductions of pro-inflammatory mediators. Given the contribution of 2-AG catabolism to eicosanoid production, it is unsurprising that several studies have reported eCBs as demonstrating pro-inflammatory roles, some examples of which include models of nephropathy (Mukhopadhyay et al., 2010a), cardiomyopathy (Mukhopadhyay et al., 2010b), and experimental dermatitis (Oka et al., 2006). Most of such pro-inflammatory effects are attributed to 2-AG and not anandamide, likely due to its considerable abundance over anandamide. However, FAAH inhibition, similarly has been found to protect against TBI-induced microglial activation (Katz et al., 2015), as too has activation of CB_2_ receptors (Amenta et al., 2012). Thus, a need exists to disentangle the potential contributions of 2-AG to pro-inflammatory processes from its role as a substrate for AA production, versus anti-inflammatory effects through cannabinoid receptors, following TBI.

Inhibition of eCB degradative enzymes has also produced decreases in TBI-induced pro-inflammatory mediators. Reductions in the expression of inducible enzymes that trigger eicosanoid production following brain injury, COX-2 enzyme (which converts free AA to prostaglandins) and iNos (which produces the free radical nitric oxide in response to cytokine signaling), are seen in response to ABHD6 inhibition (Tchantchou and Zhang, 2013) and FAAH inhibition (Tchantchou et al., 2014). Reductions in TBI-induced pro-inflammatory cytokine mRNA (Il-1β, TNFα, and IL-6) have also been found following treatment with exogenous 2-AG (Panikashvili et al., 2006). These findings seem counter-intuitive given the possibility of the rapid oxidation of 2-AG and its consequent contribution to eicosanoid production. However, exogenous 2-AG has also been shown to ameliorate TBI-induced transactivation of the nuclear factor NF-kB (linked to cytokine production) in wild type mice, but not in CB_1_ knockout mice, suggesting that CB_1_ receptors mediate the protective effects of exogenous 2-AG (Panikashvili et al., 2005).

#### Cerebrovascular Breakdown

The blood vessels which carry oxygen rich blood to the brain are lined by endothelial cells as well as astrocytes. These cells, combined with specific transport proteins and enzymes, strictly regulate movement between the general circulation and CNS extracellular fluid, and are collectively known as the BBB. TBI has been well documented in producing cerebral blood flow pathology (Kelly et al., 1997) as well as interfering with BBB integrity (Baskaya et al., 1997). Given that cannabinoids are known to exert vascular effects, producing vasodilation as well as hypotension (reviewed in Hillard, 2000b), their manipulation may hold promise as protectants against cerebrovascular damage. Below, we review studies examining the effects of cannabinoids on TBI-induced disruption of BBB integrity.

Exogenous administration of 2-AG (Panikashvili et al., 2006), as well as MAGL inhibition (Katz et al., 2015), and ABHD6 inhibition (Tchantchou and Zhang, 2013) administered post-injury protect against BBB breakdown. However, Panikashvili et al., 2006 found that the expression of proteolytic enzymes implicated in BBB breakdown were unaffected by exogenous 2-AG post-injury. These enzymes include matrix metallopeptidase-9 (MMP9) involved in extracellular matrix degradation, and tumor necrosis factor-α-converting enzyme (TACE), which cleaves membrane-bound proteins. The mechanism by which 2-AG acts as a protectant of BBB integrity following traumatic insult is yet to be resolved.

One study found that post-surgery administration of a FAAH inhibitor protected against BBB breakdown (Katz et al., 2015), suggesting that anandamide and/or other substrates of this enzyme play a protective role. While the mechanism underlying the structural protection of the BBB was not explored following TBI, anandamide has been found to decrease BBB permeability in a model of ischaemic stroke by transient receptor potential cation channel, subfamily V, member 1 (TRPV1) (Hind et al., 2015). Given that activation of TRPV1 receptors disrupts BBB integrity (Hu et al., 2005), it is possible that anandamide, as a partial agonist at TRPV1 channels (Pertwee and Ross, 2002), maybe be acting as a functional antagonist against a high efficacy endogenous agonist to produce its structurally protective effects of the cerebral microvascular endothelium. The exploration of how anandamide may be exerting its protective effects of BBB integrity may yet yield further novel targets for the treatment of TBI.

In cerebral circulation, CB_1_ receptor activation produces vasodilation. Indeed, the CB_1_ receptor antagonist rimonabant inhibited hypotension induced by endotoxin shock and hemorrhagic shock, as well as increasing survival (Varga et al., 1998). Though cannabinoids are yet to be explored in the context of TBI-induced changes in cerebral blood flow, CB_1_ receptor antagonism may prove to be a potential target for the treatment of TBI-induced hypotension.

#### Cell Structure/Remodeling

The key biological idea that structure dictates function also holds true for the neurophysiology of TBI. The shearing and tearing forces of TBI and subsequent secondary injury cascades produce changes in cell architecture, extracellular matrices, and the balance of fluid homeostasis, that impair neuronal function often both in a focal and/or diffuse manner throughout the brain (Gaetz, 2004). The use of cannabinoids has thus far been linked to protection against several of the CNS structural changes associated with TBI, with 2-AG being the most frequently studied eCB in this area.

While a traumatic insult can result in the rapid onset of cerebral oedema, exogenously administered 2-AG protects against TBI-induced oedema (Panikashvili et al., 2001, 2005). The observation that no such oedema protection was found following 2-AG administration in CB_1_ receptor^-/-^ mice (Panikashvili et al., 2005) suggests that this protection requires CB_1_ receptor activation. Changes in protein physiology have also been found to occur following TBI. Specifically, the presence of protein aggregates such as amyloid-β plaques (Johnson et al., 2010), p-tau (Goldstein et al., 2012), and TDP-43 (Smith et al., 1999), have been found within hours following TBI. These proteins are thought to accumulate from damaged axons and as a result of a disturbed balance between genesis and catabolism (Johnson et al., 2010). MAGL inhibitors decrease amyloid-β protein and its precursor molecule APP, as well as p-tau and TDP-43 (Zhang et al., 2014). MAGL inhibition also decreases astrocyte activation (Mayeux et al., 2016), while exogenous 2-AG following TBI reduces hippocampal CA-3 neuron loss (Panikashvili et al., 2001). These consistent protective effects of 2-AG across varied TBI-related structural pathologies point to its important role in maintaining cell structure and promoting remodeling.

Protective roles played by anandamide in injury-induced structural changes are yet to be ascertained. Though FAAH inhibition decreases APP expression post-injury, as well as increases synaptophysin (Tchantchou et al., 2014), a synaptic vesicle protein whose elimination impairs object recognition and spatial learning in mice (Schmitt et al., 2009). Furthermore, eCBs may not be working alone to offer protection from TBI-induced structural impairments. For example, estradiol decreased the number of TBI-induced immunoreactive astrocytes, which was inhibited by CB_1_ and CB_2_ receptor antagonists, while also increasing cerebral cortex mRNA levels of CB_2_ receptors (Lopez Rodriguez et al., 2011). These findings suggest that the regulatory activity of the eCB receptors in response to TBI may be mediated by endocrine as well as paracrine signaling mechanisms.

Traumatic brain injury is well described to increase CB_1_ and CB_2_ receptor expression, which includes disruption of diurnal rhythms of CB_1_ receptor expression (Martinez-Vargas et al., 2013). Post-injury treatment with a CB_1_ receptor antagonist reduces CB_1_ receptor expression at 6 weeks following injury (Wang et al., 2016), whereas ABHD6 inhibition produces increased CB_1_ and CB_2_ receptor expression (Tchantchou and Zhang, 2013). As such, TBI-induced increases in cannabinoid receptor expression are perhaps facilitated by 2-AG.

#### Neurogenic Pulmonary Oedema

Pulmonary complications are reported in 20–25% of TBI patients (Holland et al., 2003), and its severity is related to brain injury magnitude (Alvarez et al., 2015). The exact CNS circuits involved in NPE have yet to be identified, though a sudden rise in intracranial pressure, rapid sympathetic surge, increased systemic vascular resistance and increase in hydrostatic pressure in the pulmonary vasculature, as well as release of pro-inflammatory mediators may all contribute to interstitial pulmonary oedema formation (Brambrink and Dick, 1997). NPE rapidly occurs within hours of TBI onset in clinical populations (Alvarez et al., 2015), and within minutes in animal models (Atkinson et al., 1998), producing CNS hypoxia (Oddo et al., 2010) which further contributes to secondary injury. NPE is a much needed area of interest in the study of TBI.

While at the present time there are no studies evaluating the contributions of, or protection by, the eCB system to NPE following TBI, this may prove an interesting area of future investigation. Specifically, the lung possesses a basal tone of 2-AG (Avraham et al., 2008; Nomura et al., 2008), and recently it has been shown that resident lung macrophages express major components of the eCB system, CB_1_ and CB_2_ receptors as well as anandamide and 2-AG (Staiano et al., 2015). Furthermore, MAGL inhibition has already been found to be protective against LPS-induced acute lung injury in mice, and attenuated with CB_1_ and CB_2_ receptor antagonists (Costola-de-Souza et al., 2013).

### Treatment of Behavioral Deficits of TBI

The heterogeneous clinical presentation of TBI pathology in populations of survivors is reminiscent of its cellular and molecular pathophysiology described above. TBI patients report changes in mental health (depression, irritability, anxiety, and personality changes), sleep disturbance, post-traumatic headaches, persistent fatigue, epilepsy, learning and memory deficits (manifested also as impairments in attention and processing speed [Vakil, 2005]), and balance disorders (Stéfan et al., 2016). Most frequently investigated measures in the pre-clinical TBI literature include neurological motor, and learning and memory impairments, leaving a wide breadth of TBI clinical effects yet to be studied. Once again, components of the eCB system may become active to compensate for TBI symptomology given what is currently known of its regulatory effects within these areas, two examples being pain, and anxiety and depression (Corcoran et al., 2015).

In this section, we review what is currently known of cannabinoids in the context of their ability to alter post-traumatic animal behavior (see **Table 2**).

**Table 2:** Effect of cannabinoids on TBI-induced behavioral impairments.

Compound/mutant	Dose	Species	TBI model/severity	Effect	Receptor mediated	Reference
**Learning and memory**						
PF3845	5 mg/kg, i.p.	Mouse C57BL/6	CCI, severe	Y-maze deficit protection	CB_1_ Partial CB_2_	Tchantchou et al., 2014
JZL184	10 mg/kg, i.p.	Mouse C57BL/6	CHI, mild repetitive	MWM deficit reduction	Not evaluated	Zhang et al., 2014
WWL70	10 mg/kg, i.p.	Mouse C57BL/6	CCI, severe	Y-maze deficit protection No impact on MWM deficit	Not evaluated	Tchantchou and Zhang, 2013
**Neurological motor deficits**						
CB^1^ -/-	N/A	Mouse C57BL/6	CHI, severe	Impaired NSS score	CB_1_	Panikashvili et al., 2005
CB^1^ -/- +2-AG	N/A	Mouse C57BL/6	CHI, severe	Impaired NSS score	CB_1_	Panikashvili et al., 2005
O-1966	5 mg/kg, i.p.	Mouse C57BL/6	CCI, moderate	Rotarod deficit protection	Not evaluated	Amenta et al., 2012
Anandamide	1.25 μg/4 μL, ICV	Rat Wistar	CHI, moderate	Improved NSS score	Not evaluated	Martinez-Vargas et al., 2013
PF3845	5 and 10 mg/kg, i.p.	Mouse C57BL/6	CCI, severe	Beam-walk deficit protection	Partial CB_1_ CB_2_	Tchantchou et al., 2014
URB597	0.3 mg/kg, i.p.	Rat Sprague–Dawley	Lateral FPI, mild	No impact on NSS or NBS	Not evaluated	Katz et al., 2015
2-AG	5 mg/kg, i.v.	Mouse Sabra	CHI, severe	Improved NSS score	Not evaluated	Panikashvili et al., 2001
2-AG + 2-PG + 2-LG	1 mg/kg, i.v.	Mouse Sabra	CHI, severe	Improved NSS score	Not evaluated	Panikashvili et al., 2001
JZL184	10 mg/kg, i.p. 16 mg/kg, i.p. 16 mg/kg, i.p.	Mouse C57BL/6 rat Sprague–Dawley Rat Sprague–Dawley	CHI, mild repetitive Lateral FPI, mild Lateral FPI, mild	Improved NSS score Improved NSS and NBS score, out to 1 d Improved NSS and NBS score, out to 14 d	Not evaluated Not evaluated Not evaluated	Zhang et al., 2014 Katz et al., 2015 Mayeux et al., 2016
WWL70	10 mg/kg, i.p.	Mouse C57BL/6	CCI, severe	Improved NSS score Rotarod deficit protection	Not evaluated	Tchantchou and Zhang, 2013
**Anxiety-like behavior**						
PF3845	5 and 10 mg/kg, i.p.	Mouse C57BL/6	CCI, severe	Zero-maze anxiety-like profile protection	No CB_1_, CB_2_ reversal	Tchantchou et al., 2014
**Post-traumatic seizures**						
Rimonabant	2 mg/kg, i.p.	Rat Sprague–Dawley	Lateral FPI, severe	Protective against seizure threshold deficits Lowered seizure mortality	CB_1_	Wang et al., 2016

*Drug targets; CB_2_ receptor agonist (O-1966), FAAH inhibitor (PF3845), MAGL inhibitors (JZL184 and URB597), ABHD6 inhibitor (WWL70), and CB_1_ receptor antagonist (Rimonabant). TBI Model definitions; CCI (controlled cortical impact), CHI (closed head injury), and FPI (fluid percussion injury).*

#### Learning and Memory

Learning and memory impairments are among the most frequently reported symptoms following TBI, and are slow to recover with deficiencies reported 10 years later (Zec et al., 2001). The eCB system has been shown to play a well-documented role in memory regulation (reviewed in Mechoulam and Parker, 2011), and as such its manipulation holds considerable promise to address such a profound consequence of TBI.

Inhibition of the eCB hydrolytic enzymes FAAH (Tchantchou et al., 2014), MAGL (Zhang et al., 2014), and ABHD6 (Tchantchou and Zhang, 2013) have been shown to protect against TBI-induced memory impairments, suggesting that anandamide and 2-AG elevation post-TBI may offer protection from TBI-induced learning and memory deficits. The protective effects of 2-AG appear to be task specific, with ABHD6 inhibition showing learning and memory protection in a Y-maze task, but not a Morris water maze task. To date, only a Y-maze task has been used to evaluate the memory protective effects of FAAH inhibition, and this task-specific effect did not occur with a MAGL inhibitor. Mice are a well-used pre-clinical model organism to study the memory effects of TBI; however, they are known to perform behavioral tasks more readily, and with less error, when the task does not rely on aversive motivation (Stranahan, 2011). This attribute of mice may, in some part, contribute to the task-related differences seen between the Y-maze task (which uses exploratory behaviors associated with novelty) and the aversively motivated escape behavior necessary in the Morris water maze. Regardless, in clinical populations the most common memory process vulnerable to TBI involves difficulties applying active or effortful strategy’s in the learning or retrieval process (Vakil, 2005). Moving forward, the use of behavioral tasks able to selectively assess such frontal lobe-type memory impairments might improve the translational capacity of eCB TBI pre-clinical assessments (one such example being the Morris water maze Reversal Task, which evaluates cognitive flexibility).

#### Neurological Motor

Traumatic brain injury-induced neurological motor impairments currently represent the most frequently studied behavioral outcome measure in the TBI-cannabinoid literature. In clinical populations, neurological motor impairments seen as a result of TBI show spontaneous improvement over time, but one third of patients continue to experience neuromotor abnormalities 2 years after injury (Walker and Pickett, 2007). A variety of eCB system manipulations have thus far been found to be protective against the neurological motor deficits associated with murine models of TBI.

Both 2-AG and anandamide elevation provide protection against TBI-induced neurological motor deficits. MAGL inhibitors (Zhang et al., 2014; Katz et al., 2015; Mayeux et al., 2016), ABHD6 inhibitors (Tchantchou and Zhang, 2013), and exogenous 2-AG administration (Panikashvili et al., 2001), improve NSS in laboratory animal models of TBI. Moreover, ABHD6 inhibition also protects against TBI-induced rotarod deficits (Tchantchou and Zhang, 2013). Administration of exogenous 2-AG did not enhance NSS scores in CB_1_ receptor knockout mice subjected to TBI (Panikashvili et al., 2005), suggesting a CB_1_ receptor mechanism of action. FAAH inhibition has produced mixed findings in neurological motor tests, such as beam-walk deficit protection (Tchantchou et al., 2014) but no improvement on TBI-induced NSS deficits (Katz et al., 2015). In support of anandamide being protective against TBI-induced motor deficits, exogenous anandamide has also produced improved NSS performance (Martinez-Vargas et al., 2013). Full reversal, and partial reversal, of FAAH inhibitor mediated beam-walk deficit protection by respective CB_2_ and CB_1_ receptor antagonists (Tchantchou et al., 2014), suggest a role of both of these receptors in anandamide’s neuromotor deficit sparing effects. The involvement of the CB_2_ receptor is further supported by rotarod deficit protection from a CB_2_ receptor agonist (Amenta et al., 2012).

The role of entourage effects has also been evaluated in the area of TBI-induced neurological motor impairments. Co-release of endogenous fatty acid derivatives can potentiate 2-AG signaling, termed an entourage effect (Ben-Shabat et al., 1998; Lambert and Di Marzo, 1999; Lichtman et al., 2002). Administration of 2-AG with two related lipids that do not bind cannabinoid receptors, 2-LG and 2-PG, enhances recovery from TBI-induced NSS deficits (Panikashvili et al., 2001). Given FAAH is responsible for the degradation of various fatty acid amides in addition to anandamide (Boger et al., 2000), its various substrates may work in concert to ameliorate pathologies related to TBI. Thus any inferences drawn about anandamide through the use of FAAH inhibition needs to consider contributions of non-cannabinoid fatty acid amides.

#### Anxiety and Post-Traumatic Seizures

The signs of post-traumatic anxiety have been difficult to replicate in murine models of TBI (Tucker et al., 2016). Also, as there is a limited number of studies evaluating eCBs in this area, no definitive conclusions can be made. Thus far, only FAAH inhibition has been explored to address post-traumatic anxiety, and was found to protect against TBI-induced increases in anxiety-like behavior in mice (Tchantchou et al., 2014). This protection in the zero maze was unaffected by either CB_1_ or CB_2_ receptor antagonists, suggesting that these receptors are dispensable. Modeling post-traumatic epilepsy is time consuming and faces other challenges such as a low percentage of animals that develop epilepsy (Mazarati, 2006), however, recent models that produce consistent replication of spontaneous seizure activity following a TBI are available (Ping and Jin, 2016). Contrary to preclinical research demonstrating that the eCB system plays a protective roles against seizures (Wallace et al., 2001; Marsicano et al., 2003), a CB_1_ receptor antagonist has protected against injury-induced seizure threshold deficits as well as lowered seizure mortality (Wang et al., 2016), potentially through the disinhibition of GABAergic terminals.

This nascent body of data, suggests that eCB manipulations hold promise to treat injury-induced clinical symptoms outside of the more popular areas of learning and memory and neurological motor impairments.

## Primary Phytocannabinoids and Traumatic Brain Injury

Although currently well over one hundred phytocannabinoids have been elucidated from the *Cannabis sativa* plant (Elsohly et al., 2017), the most extensively studied of these are THC and cannabidiol (CBD). The investigation of phytocannabinoids on TBI pathology not only holds topical relevance, but also but also holds promise as potential treatment for TBI and other disorders.

Without exception, all of the experimental work reviewed and listed in **Tables 1** and **2** have used post-injury drug administration times ranging from 15 min to several days, clearly an attempt to simulate clinical intervention timing possibilities. However, clinical and pre-clinical findings provide evidence suggesting that the primary psychoactive constituent of *Cannabis sativa*, THC, is neuroprotective when administered prior to a traumatic insult. In a 3 year retrospective study of patients who had sustained a TBI, urine toxicology screen results showed decreased mortality in individuals with a positive THC screen (Nguyen et al., 2014). In two mouse models of CNS injury that yield cognitive deficits, pentylenetetrazole (an excitotoxic agent) and carbon monoxide induced hypoxic injury, prior administration of THC provided impairment protection (Assaf et al., 2011). Curiously, an extraordinarily a low dose of THC (i.e., 0.002 mg.kg^-1^) reduced injury-induced cognitive deficits in mice (Assaf et al., 2011). The authors explained this effect through the known biphasic effects of THC producing analgesia, acute hypothermia, and decreased locomotion at high doses (10 mg.kg^-1^), and producing hyperalgesia, hyperthermia, and increased locomotion at a low dose (0.002 mg.kg^-1^) (reviewed in Sarne et al., 2011). Such low dose effects of THC have been found to potentiate calcium entry into cells *in vitro* (Okada et al., 1992), increasing glutamate release, and thus may be mildly neurotoxic. Therefore, Assaf et al. (2011) hypothesized that low dose THC pre-treatment produced a pre-conditioning effect, where a mildly noxious stimulus becomes protective against a more severe subsequent insult, an effect known to occur in cardiology (Dirnagl et al., 2003) as well as cerebral ischaemia (Kitagawa et al., 1991). Moreover, the molecular signaling cascades behind cardiac and cerebral ischaemia preconditioning include activation of ERK and Akt (Dirnagl et al., 2003; Gidday, 2006), also shown to mediate the protective effects of ABHDB (Tchantchou and Zhang, 2013) and MAGL (Mayeux et al., 2016) inhibition following TBI.

Even though 80–90% of THC is excreted from individuals within 5 days of administration, the remaining slow release of lipophilic THC from lipid-storage compartments result in its long terminal half-life in plasma (Huestis, 2007). As such, individuals may experience very low plasma THC concentrations for prolonged periods after each application. Although the clinical study of TBI-induced mortality reported no data to quantify levels of THC in the THC positive individuals, the low dose THC in CNS injured mice may mimic the pharmacokinetics of THC in humans. This presumed prolonged exposure of THC due to its pharmacokinetics, as well as other potentially neuroprotective cannabinoids, such as CBD (Perez et al., 2013), may be responsible for the survival effects found in cannabis-exposed TBI patients. A finding of increased clinical relevance, is that post-conditioning (when the mildly noxious stimulus is applied after the insult) with low dose THC also produced cognitive sparing effects in mice (Assaf et al., 2011). These findings, however, remain controversial, and are yet to be replicated in animal models of TBI.

The phytocannabinoid CBD, currently being investigated in clinical trials for its seizure reduction potential in Tuberous Sclerosis Complex (Gw Research Ltd, 2016), has known anti-inflammatory properties. Although CBD does not bind CB_1_ and CB_2_ receptors, it activates the g-protein coupled receptor GPR55 (Ryberg et al., 2007), inhibits nucleoside transporter 1 (Carrier et al., 2006), inhibits sodium channels (Hill et al., 2014), and produces increased extracellular adenosine concentrations that consequently downregulate inflammatory cells through the adenosine A_2A_ receptor (Ohta and Sitkovsky, 2001; Hasko and Pacher, 2008). While there are no studies at present which have investigated the anti-inflammatory effects of CBD following TBI, CBD has reduced FosB expression following cryogenic spinal cord injury (Kwiatkoski et al., 2012), and lowered iNos expression in a mouse model of tauopathy (Casarejos et al., 2013). As such CBD may be a promising future avenue of investigation in the study of neuroinflammation in response to brain injury.

## Concluding Remarks and Future Directions

The eCB system, through release of its endogenous ligands or by changes in cannabinoid receptor constitutive activity, possesses promise in the treatment of diverse TBI pathology. An important step forward in understanding the role that the eCB system plays in TBI pathology includes not only the full characterization of ligands targeting cannabinoid receptors and eCB regulating enzymes, but also changes in cannabinoid receptors, eCB levels, and eCB regulating enzymes as a consequence of TBI. Another future area of therapeutic interest is non-CB_1_/CB_2_ receptor targets, such as TRPV1 receptors, and their potential contribution to the protective effects following TBI. Furthermore, alternative activation of CB_1_/CB_2_ receptors, such as potential entourage effects from other fatty acid derivatives, antagonism, or allosteric modulation, might impact functional selectivity and thus TBI-related outcomes also warrants further investigation. So too do the plant-derived phytocannabinoids represent an understudied yet promising group of compounds given the neuroprotective results obtained from other types of CNS injury. In particular, CBD as well as other phytocannabinoids which do not bind cannabinoid receptors, represent promising molecules to treat TBI.

To date, the only reported cannabinoid to be specifically evaluated for the treatment of TBI in patient populations is Dexanabinol, also known as HU211. While HU211 showed promise in animal models of TBI (Shohami et al., 1995), it failed to produce long term patient outcomes in one clinical trial despite some acute benefits (Knoller et al., 2002), and in a second study showed no short or long term benefits (Maas et al., 2006). Although HU211 has been described as a cannabinoid by virtue that it is an enantiomer of the potent synthetic cannabinoid agonist HU210, it does not bind or activate cannabinoid receptors. Instead, HU211acts as a non-competitive NMDA receptor antagonist (Feigenbaum et al., 1989). This therefore brings to light an important consideration of the classification of cannabinoids.

One consistently overlooked area across the study of TBI is the evaluation of the central penetration of systemically administered drugs. Pharmacological treatments will need to be assessed for their ability to cross the BBB. Also, it should be noted that TBI rapidly disrupts the BBB and lasts for three days post-injury (Baskaya et al., 1997). Furthermore, given the often biphasic nature of cannabinoid drugs, it is critical to move away from single dose pharmacology to full dose-response assessments, which may yield an increased understanding of the mechanism and potential of cannabinoids to treat TBI. Overall, the abundant and growing pre-clinical research suggests that the eCB system possesses many promising targets for new and existing drugs that may ameliorate diverse TBI pathology.

## Author Contributions

LS performed the literature review and composed the article; AL contributed to the composition of the article.

## Conflict of Interest Statement

The authors declare that the research was conducted in the absence of any commercial or financial relationships that could be construed as a potential conflict of interest.

## Abbreviations

2-AG: 2-arachidonyl glycerol
2-LG: 2-linoleoyl-glycerol
2-PG: 2-palmitoyl-glycerol
AA: arachidonic acid
ABHD6: α/β-hydrolase domain-6
ABHD12: α/β-hydrolase domain-12
AEA: anandamide
APP: amyloid precursor protein
BBB: blood brain barrier
CCI: controlled cortical impact model of TBI
CDTA: calcium-dependent transacylase enzyme
CHI: closed head injury model of TBI
COX-2: cyclooxygenase-2
cPLA2: cytosolic phospholipase A2
DAGL-α: diacylglycerol lipase-α
DAGL-β: diacylglycerol lipase-β
eCB: endocannabinoid
EPSC: excitatory post-synaptic current
FAAH: fatty acid amide hydrolase
FPI: fluid percussion injury model of TBI
LTP: long term potentiation
MAGL: monoacylglycerol lipase
NArPE: N-arachidonoyl phosphatidylethanolamine
NBS: neurological behavioral score
NMDA: N-methyl-D-aspartate
NPE: neurogenic pulmonary oedema
NSS: neurological severity score
PLA2: phospholipase A2 enzyme
PLC: phospholipase C enzyme
p-tau: hyperphosphorylated tau
ROS: reactive oxygen species
TBI: traumatic brain injury
TDP-43: TAR DNA-binding protein
THC: Δ^9^-tetrahydrocannabinol
